# Supplementary figures and images for: A network-based approach to integrate nutrient microenvironment in the prediction of synthetic lethality in cancer metabolism
Source: PLoS Comput Biol. 2022 Mar 14;18(3):e1009395. doi: 10.1371/journal.pcbi.1009395 (PMC8947600; doi:10.1371/journal.pcbi.1009395)

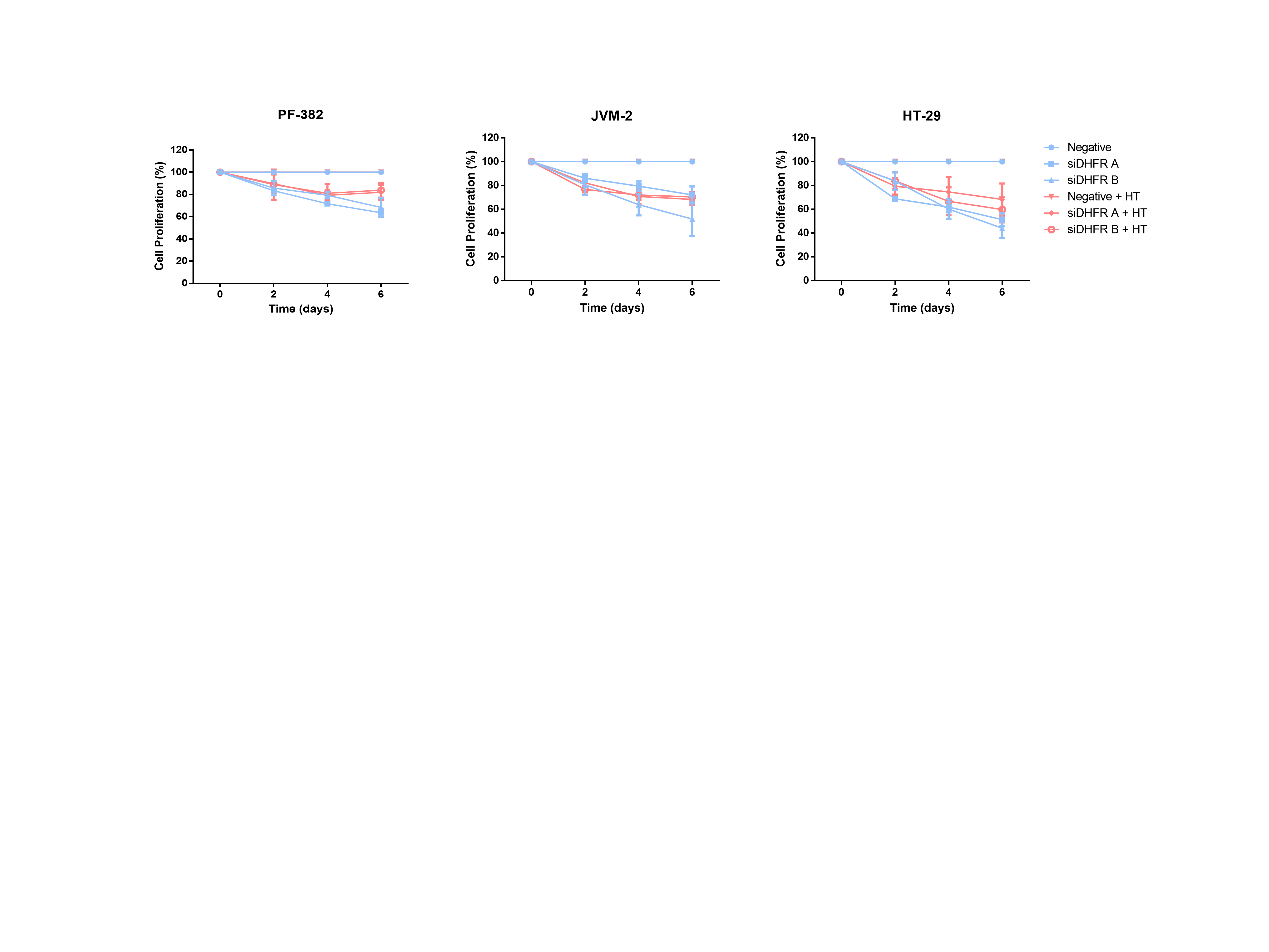

Supplement: S1 Fig — The proliferation percentage refers to cells nucleofected with a negative control siRNA. Data represent mean ± standard deviation of four experiments. (TIFF) [file pcbi.1009395.s011.tiff]

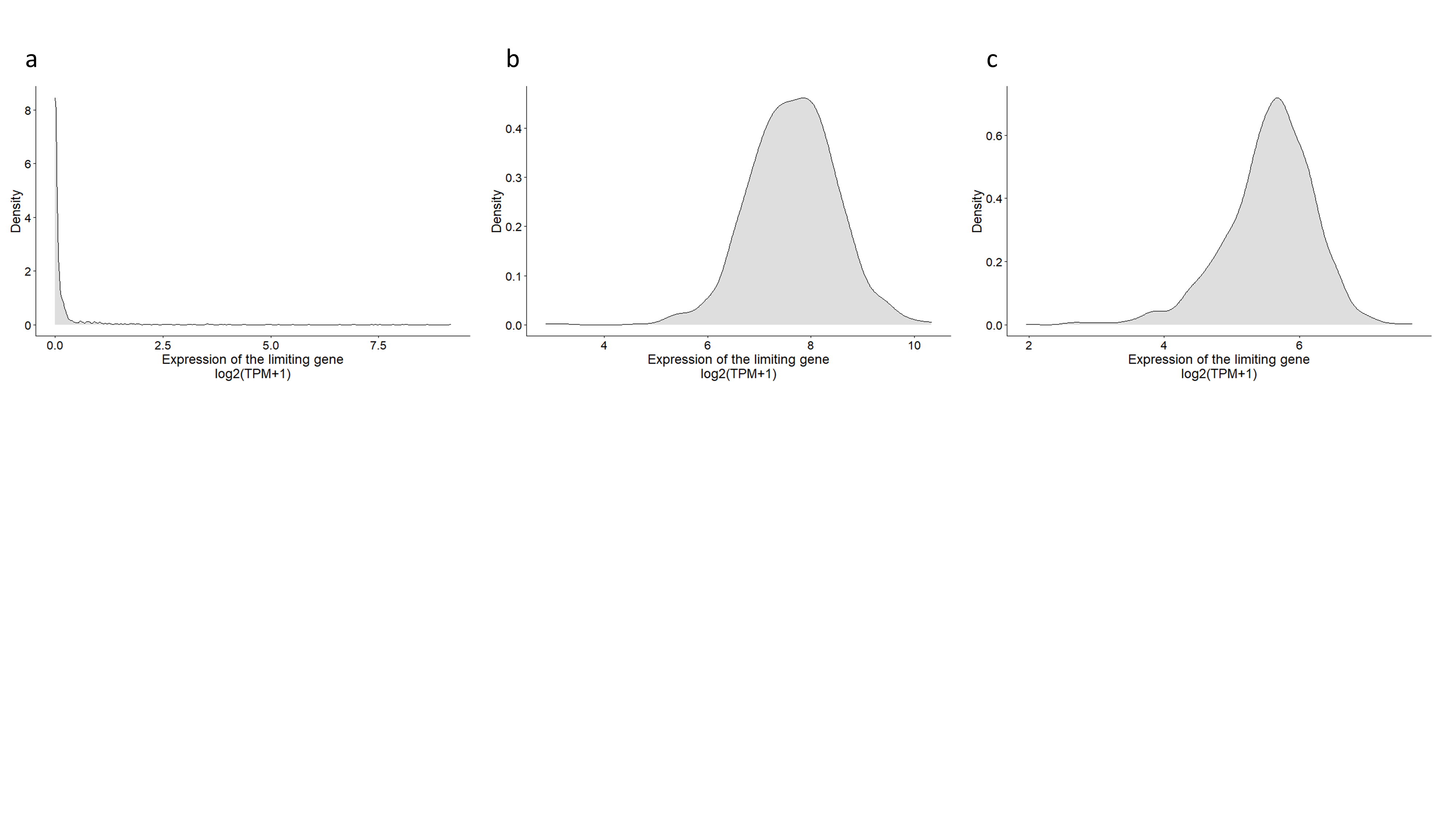

Supplement: S2 Fig — Expression level of the limiting gene in the ngMCSs involving a) L-tyrosine, b) glucose and c) choline in cancer cell lines obtained from CCLE (Ghandi et al., 2019). (TIF) [file pcbi.1009395.s012.tif]

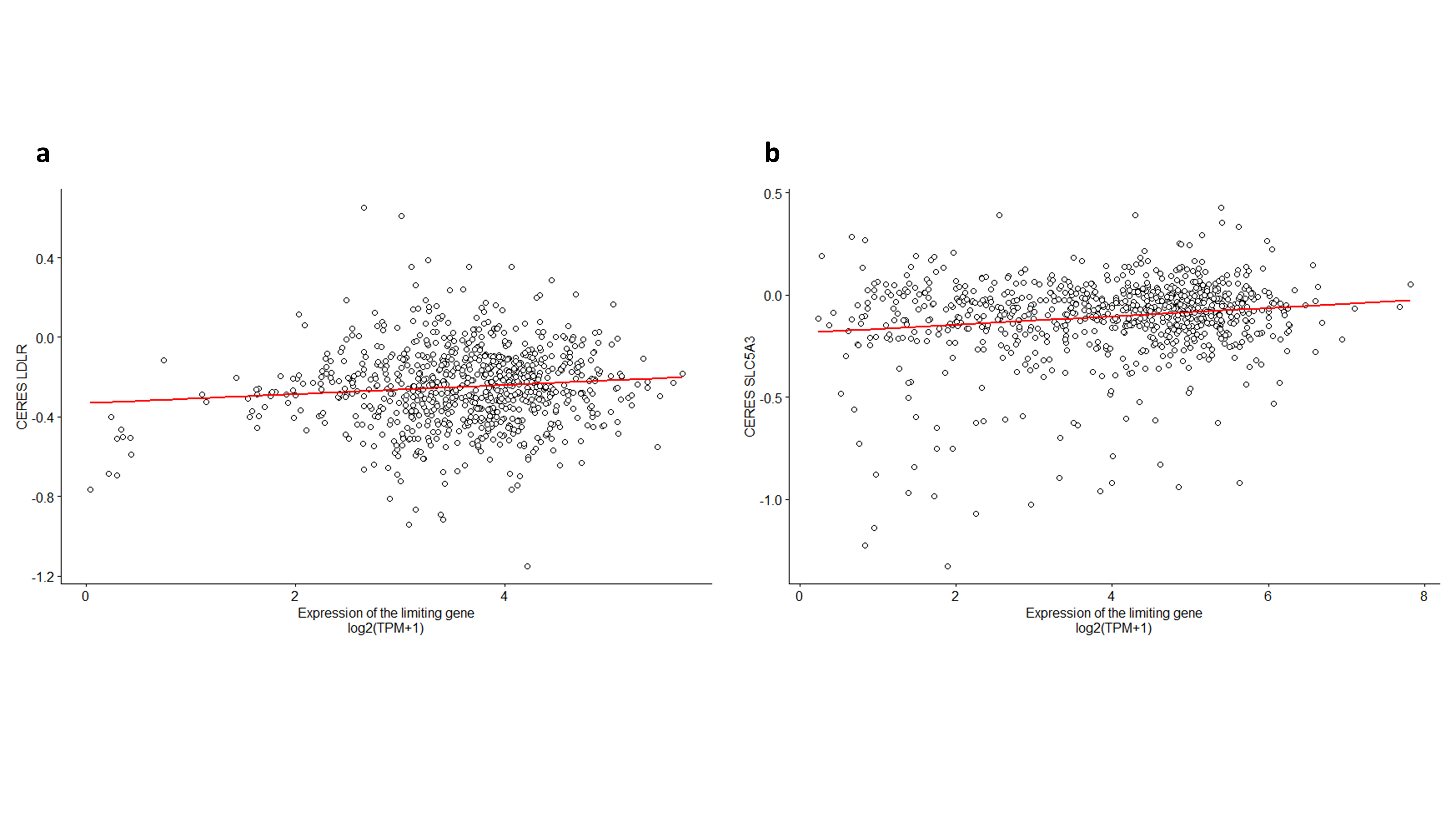

Supplement: S3 Fig — a) Dependence of the CERES essentiality score of LDLR on the expression of the limiting gene in the ngMCSs involving cholesterol. The slope of the regression line is statistically significant (p = 0.00464, r = 0.097). b) Dependence of the CERES essentiality score of SLC5A3 on the expression of the limiting gene in the ngMCSs involving myo-Inositol. The slope of the regression line is statistically significant (p = 6.67·10–6, r = 0.154). Gene expression data was obtained from CCLE (Ghandi et al., 2019) and CERES scores were obtained from the DepMap platform (Tsherniak et al., 2017). (TIF) [file pcbi.1009395.s013.tif]

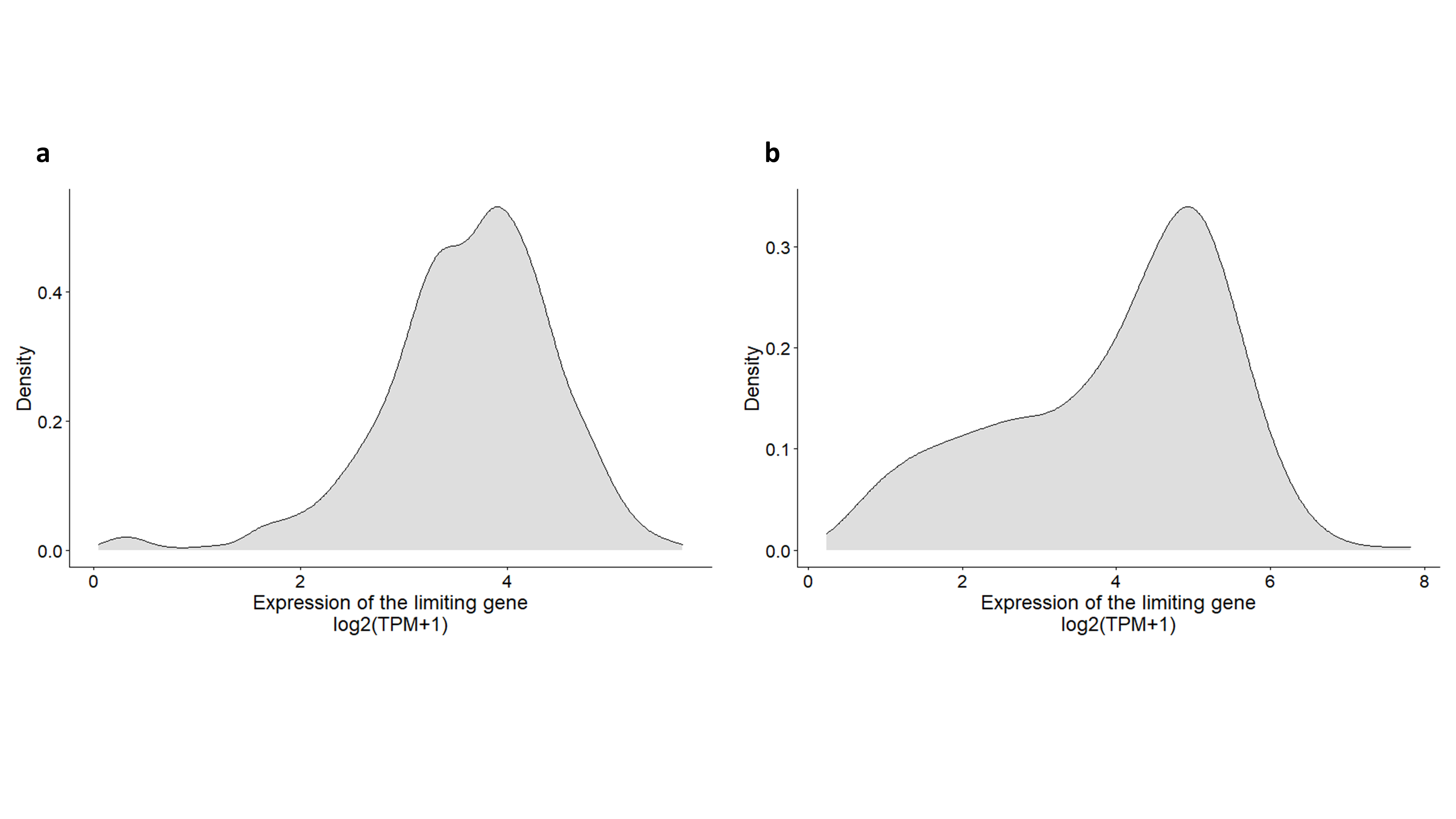

Supplement: S4 Fig — Expression level of the limiting gene in the ngMCSs involving a) cholesterol and b) myo-Inositol in cancer cell lines obtained from CCLE (Ghandi et al., 2019). (TIF) [file pcbi.1009395.s014.tif]
