# Supplementary material for: A network-based approach to integrate nutrient microenvironment in the prediction of synthetic lethality in cancer metabolism
Source: PLoS Comput Biol. 2022 Mar 14;18(3):e1009395. doi: 10.1371/journal.pcbi.1009395 (PMC8947600; doi:10.1371/journal.pcbi.1009395)
Supplement: Attachment — Submitted filename: Apaolaza et al Review.pdf [file pcbi.1009395.s015.pdf]

Title: A network-based approach to integrate nutrient environment in the prediction of synthetic lethality in cancer metabolism

Iñigo Apaolaza, Edurne San José-Eneriz, Luis V. Valcarcel, Xabier Agirre, Felipe Prósper and Francisco J. Planes

Overview and general recommendation:

Synthetic lethality is a promising general approach for discovering novel patient-specific vulnerabilities in cancer. Although there are now large amounts of gene knockout data that directly examine this question, computational modeling is still crucial to enumerate the vast number of synthetic lethality possibilities. Perhaps more importantly, such modeling, particularly when using constraints-based modeling of metabolism, can help provide mechanistic explanations for synthetic lethality, and guide development of new drug therapies.

The current paper is based on previous work that defined the concept of gene minimal cut sets (gMCS) , minimal groups of genes that must be knocked out together to attain a specific metabolic objective, such as loss of flux through the biomass reaction. The authors explore a natural extension, which is to introduce the possible absence of nutrients in the extracellular medium, through assignment of a pseudogene to nutrient exchange reactions in the human metabolic model. Although this might seem to be a very easy extension of their prior work, it leads to confirmation of some well-known nutrient-based metabolic vulnerabilities in cancer, as well as novel predictions in several different cancer types.

My overall recommendation is to publish this paper, but with some moderate revisions, mainly due to unclear statistics in Figures 3 and 4. Depending on what the actual p-values are in these figures, some conclusions may need to be revised.

Major Comments:

1. In the text, you mention that one of the siRNA lines for JVM-2 showed no effect. It is not clear to me which effect you are referring to here. Based on Figure 3C, does this mean that the original siRNA knockdown did decrease proliferation, but that addition of hypoxanthine did not rescue this decrease?

2. In general, for Figure 3B and C, it would be good to add explicit p-values to all comparisons between different groups. It is not clear often whether or not the error bars overlap between two groups, and if so, whether the overlap is significant or not.

3. I would also like to see specific p-values in as well in Figures 4C and E. This is especially important, because many of the auxotrophic subgroups, which are being compared with the prototrophs for each nutrient, are very small and may suffer from large p-values as a result. The best example of this is in the comparison of the Auxotrophic Other vs. Prototrophs in Figure 4E. The data points appear very similar to each other in these two groups. If it turns out this or any other difference is not significant, this shoould be indicated, with a discussion of why it may be so.

4. For nutrients such as thymidine and hypoxanthine that are involved in ngMCS's, tt would be good to indicate what the concentration of such nutrients typically is in body fluids. Although such measurements may not be available for the tumor microenvironment specifically, it is possible they

may have been measured in blood or other environments. If there really are no such measurements available, then this can also be stated in the text.

5. There are a few sentences whose meaning was unclear to me. I have highlighted them below, as well as my suggested revisions.

Original: Synthetic Lethality (SL) define as a type of genetic interaction where the co-occurrence of two (or more) genetic events results in cellular death, while the occurrence of either event on its own is compatible with cell viability represents a promising approach (Iglehart and Silver, 2009).

Revised: Synthetic lethality (SL), defined as a type of genetic interaction where the co-occurrence of two (or more) genetic events results in cellular death, while the occurrence of either event on its own is compatible with cell viability, represents a promising approach to develop new cancer therapies (Iglehart and Silver, 2009).

Original: Figure 1 shows an example metabolic network under two different culture mediums (environmental contexts), CM1 and CM2, where metabolite C is essential for tumor growth.

Revised: Figure 1 shows an example metabolic network under two different culture mediums (environmental contexts), CM1 and CM2. The network ultimately produces the metabolite C, which is essential to tumor growth.

Original: Thus, we focus on the other 6 nutrients (Figure 4a). (Should be just 5 nutrients?)

Revised: Thus, we focus on the other 5 nutrients (Figure 4a).

Original: All together emphasizes the importance of systematically considering the nutrient environment to target cancer metabolism via synthetic lethality approaches (Muir and Vander Heiden, 2018).

Revised: All of these results together emphasize the importance of systematically considering the nutrient environment to target cancer metabolism via synthetic lethality approaches (Muir and Vander Heiden, 2018).

6. Finally, for all ngMCS that have been identified in this paper, it would be good to plot each of them upon a pathway map of the human metabolic network. I would expect that most of them cluster in a few pathways related to processing of a particular nutrient. Conversely though, if there are any ngMCS that fall into more isolated regions of metabolism, that could highlight exciting and previously unknown associations.
