# Supplementary material for: A network-based approach to integrate nutrient microenvironment in the prediction of synthetic lethality in cancer metabolism
Source: PLoS Comput Biol. 2022 Mar 14;18(3):e1009395. doi: 10.1371/journal.pcbi.1009395 (PMC8947600; doi:10.1371/journal.pcbi.1009395)
Supplement: Attachment — Submitted filename: Response Letter.docx [file pcbi.1009395.s016.docx]

**Comments to the Authors:**

***Reviewer #1:*** *The authors have conducted an in silico study of synthetic lethality that adds nutrient availability as a dependency alongside genes. They validate one of their predictions in vitro and use published high throughput in vitro data to corroborate two additional predictions. The computational approach is a natural extension of previous work and can be expected to overlap with results involving transporter knock outs to some degree, but the method should still be of interest given the importance of the tumor micro environment for understanding differential responses to therapies. The clarity and discussion of clinical feasibility/study limitation could be further improved in a minor revision as detailed in the following specific comments.*

*Major comments:*

*The authors propose a new definition of synthetic lethality; however, it is not clear that a new definition is warranted. A downside with the proposed definition is that two different entities (genes and metabolites) are included under the same concept, although this may perhaps be alleviated by considering them druggable dependencies. The definition proposed in the abstract is not sufficiently precise, and it may perhaps be premature to propose an expansion of an existing definition in an original research article. Perhaps context dependent lethality/SL will suffice for now?*

**Response:** We appreciate the general positive comment regarding our manuscript. We also agree that additional studies may be needed to definitively modify the definition of SL, however, we believe that unlike previous studies which only consider genetic interactions for a metabolic model in a predefined growth medium our approach is fundamentally different as context-specific synthetic lethality would not sufficiently reflect the relevance of our approach to identify nutritional dependences. In any case, we have modified the Abstract and Introduction to acknowledge the reviewer comment and to clarify our new proposed concept of synthetic lethality. See the last paragraph of Page 3 in the main text:

***“These results evidence the need of a more general approach to target cancer metabolism that integrates both genetic and environmental factors. However, this has not been systematically explored in current approaches to SL in cancer metabolism. In this article, we propose a new definition of SL that that combines loss of gene functions and absence of nutrients in the environment. Our definition can help to contextualize genetic dependencies in different environmental scenarios, e.g. if extracellular uridine is absent in the microenvironment, then dihydroorotate dehydrogenase becomes lethal in leukemia. Moreover, we could reveal nutrient dependencies according to the genetic context, as the case mentioned above where the blockage of the uptake of L-asparagine is lethal in patients with loss of function of asparagine synthetase.”***

*A stronger case for the clinical relevance of context dependent SL could be made in the introduction, e.g. it is mentions that SL is a promising approach, but not how; it is mentioned that cancer could be an application of SL but it is not established that nutrient availability could be a druggable target, although in the result section it is later mentioned that depletion of certain amino acids have been used as therapy. The reverse concept of nutritional requirements to overcome lethality would also benefit from a more through introduction. How do the authors envision that nutrients could be controlled in the micro environment? Or if depletions are systemic, what side-effects could be anticipated and tolerable?*

**Response:** We appreciate the comment made by the reviewer. We have extended the Introduction section to highlight the clinical relevance of considering tumor microenvironment in developing metabolic targets in cancer. In particular, we added the following paragraph in Page 3 in the main text:

***“Despite these promising results, the application of SL to cancer metabolism has still different challenges. One of them is the integration of tumor microenvironment in the prediction of SL (Rancati et al., 2018), since metabolic genetic interactions and nutritional availability are intertwined to support cellular proliferation. On the one hand, the presence/deprivation of certain nutrients in the environment could modify the metabolic landscape and explain tumor resistance/sensitivity to metabolic targets. For example, it was recently reported that the supplementation of uridine rescues the anti-leukemic effect of dihydroorotate dehydrogenase inhibition (Sykes et al., 2016). On the other hand, targeting nutrient dependencies of tumor cells is an emerging topic in cancer research (Li et al., 2019), and they are directly connected to the genetic variations of tumor cells but disconnected from the synthetic lethality literature. A paradigmatic example is the dependency on extracellular L-asparagine in samples with loss of asparagine synthetase (Lazarus et al., 1969). In fact, L-asparagine depletion via asparaginase administration is an approved therapy for acute lymphoblastic leukemia (Narta et al., 2007).”***

In addition, a paragraph has been added in the Discussion section describing different strategies to control the microenvironment and their potential side effects:

***“Current therapeutic approaches to control the level of nutrients in the tumor microenvironment include nutrient-degrading enzymes and custom diets. The intravenous administration of L-asparaginase enzyme to acute lymphoblastic leukemia patients is one successful example. This enzyme, synthetized from E. coli, involves relevant toxic effects; however, they are nonfatal, manageable, and reversible (Aldoss and Douer, 2020), which is encouraging for further developments. On the other hand, the effectiveness of custom diets in cancer has been successfully tested in mouse models (Maddocks et al., 2017). However, their application to cancer patients is an open question and further clinical studies are required to demonstrate their benefit.”***

*The reader would benefit from a more thorough description of the cut set method in the background. Integer programming is an NP-complete problem, so is the method guaranteed to find all solutions, or will it only find a subset? The method section mentions a 60 second search cut-off, if this was increased by several orders of magnitude, would more and/or different cut sets be expected? For linear pathways where all reactions have the same effect, which one will be selected by the model? Will this choice be consistent or stochastic? Why are gMCS of size 1 discarded? How does the gMCS method compare to other approaches for SL using genome scale models, e.g. Agren 2014.*

**Response:** As suggested by the reviewer, we have included a new sub-section, called “The genetic Minimal Cut Set approach”, in the Methods section, where different technical settings in our previously developed algorithm are described.

In addition, gMCSs of size 1 were not considered in this study since they are essential for all cell types, and our particular interest is focused on cancer-specific essential genes.

Finally, we think that the comparison of our gMCS approach with other methods is out of the scope of this manuscript. In our previous paper (Apaolaza et al. 2017), we carried out a side-by-side comparison with outstanding methods at that time, finding a superior performance of our gMCS approach. We are currently preparing a new manuscript with an updated comparison of our gMCS approach with other methods, particularly for predicting essential genes. However, we would like to emphasize that the objective of this manuscript is to present and apply the new concept of nutrient-genetic Minimal Cut Sets (ngMCSs).

*The method enables identification of more simultaneous knockouts than brute force methods, but is it reasonable to expect that 10+ dependencies could be targeted clinically? Perhaps it is reasonable to say that the window of relevance for this method lies between around 3-5 KOs, i.e. where brute force is infeasible (>2 dependencies) yet the results are clinical is feasible (<~5). Would it be feasible to introduce an upper bound on number of elements in the cut-set? Alongside the elegant case studies performed in this paper, it seems like the authors should be in a good position to perform a more global analysis, how many clinically feasible SL options are there? How many metabolites are in these sets? Are the 8 nutrients that were identified the only ones that could be SL? Could the analysis in figure 4 be done more systematically? e.g. scattering expression of dependencies against CERES scores across multiple conditions?*

**Response**: We may easily fix an upper bound on the number of elements in our solutions. However, the reviewer should consider that we may have one gMCS of size 10 where 9 genes are not expressed and other gMCS of size 3 where 2 genes are not expressed. In our opinion, both gMCSs are equally interesting and lead to an essential gene in the molecular context considered. Thus, in our opinion, clinically feasible SL depends on the length but also on the context considered. For this reason, we prefer not to restrict the length of the solutions.

In this direction, it would be very interesting to assess whether essential genes coming from large gMCSs have the same confidence than short gMCSs, since large gMCSs could accumulate more errors from the reference metabolic networks or more easily involve regulatory mechanisms. However, we feel again this is out of the scope of the current manuscript.

On the other hand, we conducted a more global analysis of ngMCSs under RPMI growth medium, supplemented with FBS. As now noted in the Results section, we identified 52 ngMCSs (detailed in Supplementary Data 2), which involve 19 nutrients. In particular, we added the following paragraph in Page 12 of the main text:

***“We identified 52 ngMCSs (Supplementary Data 2), which involve 19 nutrients. Neglecting essential nutrients under RPMI growth medium and ngMCSs involving more than one nutrient, we have 8 nutrients: L-asparagine, L-arginine, cholesterol, choline, D-glucose, L-glutamine, myo-Inositol and L-tyrosine.”***

In addition, as suggested by the reviewer, we repeated Figure 4 considering all cell lines included in DepMap, detailed in Supplementary Figure 3, finding a positive and significant linear association in both cholesterol and myo-Inositol.

***Supplementary Figure 3.*** *a) Dependence of the CERES essentiality score of LDLR on the expression of the limiting gene in the ngMCSs involving cholesterol. The slope of the regression line is statistically significant (p=0.00464). b) Dependence of the CERES essentiality score of SLC5A3 on the expression of the limiting gene in the ngMCSs involving myo-Inositol. The slope of the regression line is statistically significant (p=6.67·10-6). Gene expression data was obtained from CCLE (Ghandi et al., 2019) and CERES scores were obtained from the DepMap platform (Tsherniak et al., 2017).*

*The result of nutrient availability can be expected to largely overlap with KO of the corresponding nutrient transporter, this is even the premises for figure 2, it may be useful to discuss when the method can be expected to give a different result and/or if this is mostly for analytical convenience.*

**Response:** The comment is pertinent. We have included a paragraph in the Discussion section clarifying that targeting nutrient dependencies do not necessarily overlap with the knockout of the corresponding transporters. In particular, we explain that transporters are not specific in many cases and could accept several nutrients. Moreover, nutrients typically have different transport systems, which can be more difficult to target.

Note here that myo-Inositol and cholesterol dependencies were assessed with one of the transporters previously recognized in the literature as relevant for their uptakes; however, this is not the optimal experiment. The optimal experiment would be to deplete myo-Inositol and cholesterol from the growth medium, as done in other works (Li et al., 2019). As the availability of large-scale studies evaluating the effect of nutrient depletions in cancer cell lines is lacking, the type of validation for our predictions is currently limited.

*A more through discussion on the requirements and assumptions of the method would be useful. The method depends on a static biomass equation. But in the living cell biomass is not static. This can lead to situations where a reaction is computationally identified as essential, but not necessarily biologically essential, e.g. glycogen is an energy storage molecule that cells likely can do without, e.g. some cancer cell lines have undetectable glycogen levels while others have elevated levels (Yates 2016). The method mentions different curation efforts to the model, are these anticipated to affect the results? If so it would be appropriate to mention that they have been performed in the main text and discuss in which way the results may be affected? It would perhaps also be relevant to reproduce the results in another GEM, e.g. Human1 (Robinson 2020), this model encourages users to report annotation errors and regularly updates the model. The minimal cut sets the method could be expected to disfavor knocking out reactions with multiple isozymes over knocking out two completely different reactions, however, it is conceivable that a single drug would block all isozymes (as is the case for MTX in this study).*

**Response:** We are currently conducting a separate study to elucidate whether all tumors require the same metabolites of the biomass reaction. The reviewer is right: different tumors have different degrees of dependency with respect to the metabolites in the biomass equation. The question is really interesting, but too complicated to be addressed in this manuscript.

On the other hand, adapting our gMCS approach to Human1 is not trivial, since they incorporate different metabolic tasks aside from the biomass equation. In the different manuscript that we are preparing mentioned above, this is directly addressed. In addition, as the errors reported in Supplementary Table 5 were also found in Human1, following the reviewer’s recommendation, we submitted an issue to Human1 with our proposed changes.

*A discussion of the assumption about growth medium may also be warranted. In this study the growth medium was modeled as the components of RPMI, however the cell cultures are grown with 10% FBS that contains a large number of metabolites, could this be expected to influence the results? Additionally, the HT-29 cells were grown in McCoy medium, it may be good to mention if there are any differences in composition between these media that may be of relevance, in particular since HT-29 cells were observed to react differently. In this study degradation of macromolecules is neglected for computational convenience, but could it be biologically feasible for cells to meet their amino acid needs by protein uptake and subsequent degradation?*

**Response:** The reviewer is correct. In fact, our results were obtained with RPMI supplemented with 10% FBS in the growth medium. This has been clarified in the Methods section (see Page 18).

In addition, the fixed growth medium conditions used in our analyses are described in Supplementary Data 2 (Supplementary Tables 1-3).

With respect to HT-29 cells, the differences in composition between RPMI and McCoy’s media are irrelevant for our computational analysis. This has been also reflected in the Results section (1s paragraph, Page 10).

We agree with the reviewer in that it could be biologically feasible for cells to meet their amino acid needs by protein uptake and subsequent degradation. However, as these processes are not correctly modeled in Recon3D, we decided to exclude them from the analysis. As this does not affect our hypotheses related with cholesterol and myo-Inositol, we decided to keep the analysis as previously presented.

*The authors use a cutoff of 1 TPM to consider a gene active. While this is a common practice, there are many intermediate steps between gene expression and flux, i.e. lower expression does not imply lower flux when comparing two different genes. The relative gene expression between cell lines and contexts may sometimes be more relevant to gauge activity. The authors discuss “the most limiting gene” but it is not guaranteed that the gene with lowest expression is most limiting. For L-tyrosine the text mentions lowly expressed ngMCS, however, no data is provided to support this claim. The authors report expression data in figure 4 that suggest a bimodal distribution of expression, but cell lines with intermediate expression have been removed. It is of interest to see the distribution of the intermediate genes, such information can be provided as supplementary material.*

**Response:** Following the reviewer’s comment, we have included in the Supplementary Material a similar analysis to one shown in Figure 4 but considering all cell lines included in DepMap (Supplementary Figures 3-4), avoiding the cutoff of 1 TPM. The results are similar and statistically significant. This has been indicated in the main text. In particular, we added the following paragraph in Page 15 of the main text:

***“For completeness, a similar analysis about cholesterol and myo-Inositol dependencies was done considering all cell lines included in DepMap. We found a statistically significant linear association between SLC5A3 CERES scores and the expression of ISYNA1, as well as between LDLR CERES score and the expression of the partner gene of cholesterol with the highest expression among different ngMCSs (Supplementary Figure 3). In both cases, the distribution of gene expression data suggests a bimodal structure (Supplementary Figure 4), which justifies the selection of cell lines included in Figure 4.“***

***Supplementary Figure 4.*** *Expression level of the limiting gene in the ngMCSs involving a) cholesterol and b) myo-Inositol in cancer cell lines obtained from CCLE (Ghandi et al., 2019).*

With respect to L-tyrosine, we found one ngMCS: {L-tyrosine, PAH}. As the expression of PAH (phenylalanine hydroxylase) is less than 1 TPM in 1273 out of 1379 cell lines considered in CCLE, L-tyrosine is an essential nutrient in most cell lines, being discarded for further analysis. The opposite occurs with glucose and choline, where their associated genes in ngMCSs show consistent expression (> 1 TPM) in all cell lines. This has been mentioned in Results section (see Page 12 of the main text) and illustrated in Supplementary Figure 2.

***Supplementary Figure 2.*** *Expression level of the limiting gene in the ngMCSs involving a) L-tyrosine, b) glucose and c) choline in cancer cell lines obtained from CCLE (Ghandi et al., 2019).*

*If a claim is made about difference between conditions, e.g. in figure 3 and 4, then it is convention to perform a statistical test on the data, e.g. and show the p value.*

**Response:** P-values have been represented in Figures 3 and 4, and the statistical test performed clarified in the legends.

*Minor comments:*

*The abstract makes claims of novelty. This is often considered redundant in original research articles.*

**Response:** The word has been deleted from the abstract, as recommended by the reviewer.

*The abstract mentions synthetic lethality (SL), but it is not explained.*

**Response:** We have included in the abstract a formal definition of synthetic lethality.

*The “tumor environment”, is more commonly referenced to as “tumor micro environment”.*

**Response:** We have updated the term throughout the manuscript.

*Order of tabs in supplementary data 2 is different than in the index.*

**Response:** Thanks. Corrected.

*Abbreviation “ngMCSs” used in Figure 1 before being defined.*

**Response:** We have moved gMCSs and ngMCSs to a new panel in Figure 1 (Figure 1g). In this way, the term ‘ngMCSs’ in Figure 1 is not used before being defined in the main text.

*Due to different international standards, it is common to give the base of the logarithm in subscript (e.g. Log10) if it is not the natural logarithm (e.g. figure 3).*

**Response:** We have given the base of the logarithm in Figure 3d (Log10), as required by the reviewer.

*The source of data should be made clear in the legend of figure 4.*

**Response:** We have added a sentence in the legend of Figure 4 to clarify the source of data used.

*The experimental basis of the CERES score could be explained more clearly.*

**Response**: We have added a paragraph in the Results section clarifying the concept of CERES score.

*According to methods proliferation was measured at day 0, 2, 4, and 6, but only day 6 is reported in figure 3c, this data should perhaps be made available as supplementary data.*

**Response**: Full details of proliferation studies after DHFR silencing with and without hypoxanthine and thymidine was provided as supplementary material. See Supplementary Figure 1 below.

***Supplementary Figure 1.*** *Proliferation of PF-382, JVM-2 and HT-29 cell lines nucleofected with siRNAs targeted to DHFR gene in presence or absence of Hypoxanthine-Thymidine was studied by MTS at day 2, 4 and 6 after nucleofection. The proliferation percentage refers to cells nucleofected with a negative control siRNA. Data represent mean ± standard deviation of four experiments.*

*The literature review of DHFR knock out in the methods section is not a method and should perhaps be moved to introduction or results.*

**Response**: The purpose was not to conduct a literature review, but to justify how we modelled the knockout of DHFR in our simulations. For this reason, we decided to leave it in Methods section.

Lethality is taken to mean a blocked biomass reaction, but method also mentions “removal disrupts the flux through the biomass reaction”, which is weaker than blocking, if there is a distinction it should be clarified.

**Response**: There is no distinction. The reviewer is correct. We have substituted the word ‘disrupt’ by ‘block’.

Overall the language is acceptable, however several instances of reversed word order suggest that the manuscript could benefit from proofreading by a native speaker.

**Response:** We have reviewed the manuscript with a native speaker, as suggested by the reviewer.

References

Agren, R., Mardinoglu, A., Asplund, A., Kampf, C., Uhlen, M., & Nielsen, J. (2014). Identification of anticancer drugs for hepatocellular carcinoma through personalized genome-scale metabolic modeling. Molecular systems biology, 10(3), 721.

Abstract 1040: Differential levels of glycogen in breast cancer cell lines: A potential new target Joel A. Yates, Megan Altemus, Zhifen Wu, Michelle L. Wynn and Sofia D. Merajver Cancer Res July 15 2016 (76) (14 Supplement) 1040;

Robinson JL, Kocabaş P, Wang H, Cholley PE, Cook D, Nilsson A, Anton M, Ferreira R, Domenzain I, Billa V, Limeta A, Hedin A, Gustafsson J, Kerkhoven EJ, Svensson LT, Palsson BO, Mardinoglu A, Hansson L, Uhlén M, Nielsen J. An atlas of human metabolism. Sci Signal. 2020 Mar 24;13(624):eaaz1482.

***Reviewer #2:*** *Synthetic lethality is a promising general approach for discovering novel patient-specific vulnerabilities in cancer. Although there are now large amounts of gene knockout data that directly examine this question, computational modeling is still crucial to enumerate the vast number of synthetic lethality possibilities. Perhaps more importantly, such modeling, particularly when using constraints-based modeling of metabolism, can help provide mechanistic explanations for synthetic lethality, and guide development of new drug therapies.*

*The current paper is based on previous work that defined the concept of gene minimal cut sets (gMCS) , minimal groups of genes that must be knocked out together to attain a specific metabolic objective, such as loss of flux through the biomass reaction. The authors explore a natural extension, which is to introduce the possible absence of nutrients in the extracellular medium, through assignment of a pseudogene to nutrient exchange reactions in the human metabolic model. Although this might seem to be a very easy extension of their prior work, it leads to confirmation of some well-known nutrient-based metabolic vulnerabilities in cancer, as well as novel predictions in several different cancer types.*

*My overall recommendation is to publish this paper, but with some moderate revisions, mainly due to unclear statistics in Figures 3 and 4. Depending on what the actual p-values are in these figures, some conclusions may need to be revised.*

*Major Comments:*

*In the text, you mention that one of the siRNA lines for JVM-2 showed no effect. It is not clear to me which effect you are referring to here. Based on Figure 3C, does this mean that the original siRNA knockdown did decrease proliferation, but that addition of hypoxanthine did not rescue this decrease?*

**Response:** We would like to thank the reviewer for the comments to improve the readability of the manuscript. We have rephrased the text to clarify that one of the siRNAs designed to silence DHFR decreased the proliferation of JVM-2, but the addition of hypoxanthine and thymidine did not rescue this decrease (see Page 10).

*In general, for Figure 3B and C, it would be good to add explicit p-values to all comparisons between different groups. It is not clear often whether or not the error bars overlap between two groups, and if so, whether the overlap is significant or not.*

**Response:** In this new version of the manuscript, we completed *in-vitro* experiments to have at least 3 replicates for each case considered and added statistical details to the different comparisons in Figures 3b-c (see below). It can be observed that the addition of hypoxanthine and thymidine rescued the proliferation under exposure to MTX in all cases. In the case of DHFR silencing, we found statistically significant differences in 3 out of 6 cases and almost significant differences in 2 out of 5 cases (see Figure 3c). This has been indicated in the main text (Page 10).

***Figure 3. In-vitro experimental validation of DHFR inhibition with hypoxanthine and thymidine in the growth medium.*** *b) Proliferation of PF-382, JVM-2 and HT-29 cell lines treated with different doses of MTX for 96h in presence or absence of Hypoxanthine-Thymidine. Data represent mean ± standard deviation of four experiments. c) Proliferation of PF-382, JVM-2 and HT-29 cell lines nucleofected with siRNAs targeted to DHFR gene in presence or absence of Hypoxanthine-Thymidine was studied by MTS at day 6 after nucleofection. The proliferation percentage refers to cells nucleofected with a negative control siRNA. Data represent mean ± standard deviation of at least three experiments. All p-values (p) were determined by one-tailed Wilcoxon tests. Abbreviations: MTX: methotrexate; HT: Hypoxanthine-Thymidine.*

I would also like to see specific p-values in as well in Figures 4C and E. This is especially important, because many of the auxotrophic subgroups, which are being compared with the prototrophs for each nutrient, are very small and may suffer from large p-values as a result. The best example of this is in the comparison of the Auxotrophic Other vs. Prototrophs in Figure 4E. The data points appear very similar to each other in these two groups. If it turns out this or any other difference is not significant, this should be indicated, with a discussion of why it may be so.

**Response:** Specific p-values have been included in the different comparisons shown in Figures 4c and 4e, and the statistical test performed clarified in the legend. In general, the claims previously presented in our manuscript remain valid, as mentioned in Page 13-14 of the text.

***Figure 4. Extracellular nutrient dependences of in vitro cancer cell lines.*** *b) Expression levels of the most limiting gene in the ngMCSs associated with Cholesterol in 18 auxotrophic and 18 prototrophic cell lines. Auxotrophic cell lines were defined as those where the most limiting gene has an expression level below 1 TPM. For comparison, we took the same number of prototrophic cell lines; c) CERES scores for LDLR in the cell lines shown in b). The more negative CERES scores are, the more cellular proliferation will be decreased; d) Expression levels of the most limiting gene in the ngMCSs associated with Myo-Inositol in 45 auxotrophic and 45 prototrophic cell lines. e) CERES scores for SLC5A3 in the cell lines shown in d). Abbreviations: ‘Aux.’ refers to auxotrophic cell lines, e.g. Aux. ALL refers to auxotrophic ALL cell lines. Note here that in panels (b)-(d) gene expression data was obtained from CCLE (Ghandi et al., 2019) and in panels (c)-(e) CERES scores were obtained from the DepMap platform (Tsherniak et al., 2017). The significance of the difference in LDLR and SLC5A3 CERES scores in the auxotrophic groups against the corresponding prototrophic group was assessed via a one-tailed Wilcoxon test.*

*For nutrients such as thymidine and hypoxanthine that are involved in ngMCS’s, it would be good to indicate what the concentration of such nutrients typically is in body fluids. Although such measurements may not be available for the tumor microenvironment specifically, it is possible they may have been measured in blood or other environments. If there really are no such measurements available, then this can also be stated in the text.*

**Response:** As suggested by the reviewer, we have indicated in the main text that previous studies in the literature showed a relevant plasma concentration of thymidine and hypoxanthine in cancer patients (see at the end of Page 8 of the main text). We appreciate the comment of the reviewer because their presence in plasma of cancer patients gives more relevance to the study accomplished.

*There are a few sentences whose meaning was unclear to me. I have highlighted them below, as well as my suggested revisions.*

*Original: Synthetic Lethality (SL) define as a type of genetic interaction where the co-occurrence of two (or more) genetic events results in cellular death, while the occurrence of either event on its own is compatible with cell viability represents a promising approach (Iglehart and Silver, 2009).*

*Revised: Synthetic lethality (SL), defined as a type of genetic interaction where the co-occurrence of two (or more) genetic events results in cellular death, while the occurrence of either event on its own is compatible with cell viability, represents a promising approach to develop new cancer therapies (Iglehart and Silver, 2009).*

**Response:** Done.

*Original: Figure 1 shows an example metabolic network under two different culture mediums (environmental contexts), CM1 and CM2, where metabolite C is essential for tumor growth.*

*Revised:* *Figure 1 shows an example metabolic network under two different culture mediums (environmental contexts), CM1 and CM2. The network ultimately produces the metabolite C, which is essential to tumor growth.*

**Response:** Done.

*Original: Thus, we focus on the other 6 nutrients (Figure 4a). (Should be just 5 nutrients?)*

*Revised: Thus, we focus on the other 5 nutrients (Figure 4a).*

**Response:** Correct. Updated.

*Original: All together emphasizes the importance of systematically considering the nutrient environment to target cancer metabolism via synthetic lethality approaches (Muir and Vander Heiden, 2018).*

*Revised: All of these results together emphasize the importance of systematically considering the nutrient environment to target cancer metabolism via synthetic lethality approaches (Muir and Vander Heiden, 2018).*

**Response:** Thanks again.

*Finally, for all ngMCS that have been identified in this paper, it would be good to plot each of them upon a pathway map of the human metabolic network. I would expect that most of them cluster in a few pathways related to processing of a particular nutrient. Conversely though, if there are any ngMCS that fall into more isolated regions of metabolism, that could highlight exciting and previously unknown associations.*

**Response:** The comment of the reviewer is really interesting. However, there are a number of difficulties to conduct the mapping on the human metabolic network. First, ReconMaps, the visualization tool associated with Recon3D, currently accepts metabolites but not reactions and genes. Second, the matching between Recon3D and KEGG, for example, is not complete and this limits the visualization of our ngMCSs in standard pathway maps.

One possible solution is to extract the metabolic subsystems annotated in Recon3D to the exchange reactions and genes involved in our ngMCSs. However, we may have genes involving too many reactions and this may complicate the interpretation. A better solution would be to calculate Minimal Cut Sets (MCSs) within the reactions associated to the genes (and exchange reactions) involved in our ngMCSs. The difference between MCSs and gMCSs is that the former considers reaction deletions and the latter gene deletions. This would reduce the number of reactions to consider in our ngMCSs and allows us to identify which reactions are involved in each ngMCS.

For example, consider the following ngMCS: {LPL, MVK, Cholesterol}. The gene LPL involves 16 reactions in Recon3D, while MVK 2 reactions. Once we search for MCSs in the knockout search space involving these 18 reactions plus the exchange reaction of cholesterol, we obtain the following solution:

| **rxns** | **rxnNames** | **subSystems** |
| --- | --- | --- |
| MEVK1x | Mevalonate Kinase (ATP) | Cholesterol metabolism |
| MEVK1c | Mevalonate Kinase (ATP) Cytosol | Squalene and cholesterol synthesis |
| HMR_0013 | Lipoprotein Lipase | Exchange/demand reaction |
| HMR_0014 | Lipoprotein Lipase | Exchange/demand reaction |
| EX_chsterol[e]_b | Exchange of Cholesterol | Exchange/demand reaction |

, where **rxns**, **rxnNames** and **subSystems** are the reaction code, reaction name and associated subsystem provide in Recon3D.

It is interesting to see that we do not need all the 19 reactions to block the biomass, but with 5 of them is sufficient. In addition, in this particular MCS, the reaction name seems more informative than subsystems, and it provides a better picture of metabolic processes implied. We found the same pattern with other ngMCSs.

Despite these promising results, we feel that this approach requires further work to correctly answer the question posed by the reviewer. Overall, we think that the question of visualizing and providing functional interpretation to ngMCSs/gMCSs entails sufficient complexity to be quickly addressed here. Thus, as the aim of our work is to emphasize the importance of nutrient microenvironment and present a novel approach for the more efficient identification of potential metabolic targets in cancer, we prefer to leave this question aside for the moment.

***Reviewer #3:*** *Apaolaza et al presented a computational biology application to predict synthetic lethal by utilizing the gMCSs identification method they have previously developed. Prediction of synthetic lethality is highly important for cancer treatment, which is very appreciate. This manuscript is based on rational computational consideration and supported by in vitro experiments. However, I have a few major concerns:*

*Synthetic lethal should be computed in disease specific context. The full RECON3D metabolic network contains all potential metabolic reactions, but not all of them exist in one disease condition. As the authors described in Fig 1, different cancers may have different metabolic variations that may lead to varied synthetic lethal. It is unclear in computing the lethality of DHFR inhibition, which disease context were considered. For the three validation cell lines, why their gene expression or mutation data were not utilized in the computation. In addition, even there is limited novelty of the gMCSs detection in this work, the computational approach including input data should be detailed.*

**Response:** Under RPMI growth medium, DHFR is essential for any situation, according to Recon3D. This has been also experimentally observed in a large number of cell lines, according to DepMap, including the ones shown in Figure 3 (PF-382, JVM-2, HT-29).

On the other hand, when thymidine and hypoxanthine are added to the growth medium, DHFR is not essential anymore, since the alternative pathways described in Figure 2 become active. We checked with gene expression data that the enzymes involved in these alternative pathways are highly expressed (SLC29A2, TK1 and HPRT1) (see Supplementary Table 1).

Finally, we have extended the description of our computational approach to calculate gMCSs and ngMCSs. See the new sub-section “The genetic Minimal Cut Set approach” in Methods section (see Page 19).

*A rigorous computational definition of ngMCSs is needed.*

**Response:** We have included a more rigorous definition of ngMCSs. This is included in a new sub-section in the Results section entitled “Extending genetic MCSs into nutrient-genetic MCSs” (see Page 7).

*It is unclear how gene expression data were utilized in the computation of ngMCSs and if the method considers metabolic flux.*

**Response:** We have clarified in the Results section that gene expression data is not used for the computation of both gMCSs and ngMCSs, which are structural properties of the uncontextualized reference metabolic network. Once they are calculated, we can map gene expression data and information about the presence of nutrients in the microenvironment to elucidate genetic or environmental dependencies. This can be found in the sub-section “Extending genetic MCSs into nutrient-genetic MCSs” (see 1^st^ paragraph of Page 8).

Note here that, as other algorithms in the field of constraint-based modeling, we assume metabolic fluxes are in steady state. However, our approach does not require a quantitative measure of fluxes. gMCSs and ngMCSs are structural properties of the metabolic network under consideration.

*Figure legends are too small.*

**Response:** We have increased the font size of the legends to make them easier to read.

**Have the authors made all data and (if applicable) computational code underlying the findings in their manuscript fully available?** The [PLOS Data policy](https://journals.plos.org/ploscompbiol/s/materials-and-software-sharing) requires authors to make all data and code underlying the findings described in their manuscript fully available without restriction, with rare exception (please refer to the Data Availability Statement in the manuscript PDF file). The data and code should be provided as part of the manuscript or its supporting information, or deposited to a public repository. For example, in addition to summary statistics, the data points behind means, medians and variance measures should be available. If there are restrictions on publicly sharing data or code —e.g. participant privacy or use of data from a third party—those must be specified.

Reviewer #1: **No:** According to methods proliferation was measured at day 0, 2, 4, and 6, but only day 6 is reported in figure 3c, this data should perhaps be made available as supplementary data.

Reviewer #2: Yes

Reviewer #3: Yes

**References**

Aldoss,I. and Douer,D. (2020) How I treat the toxicities of pegasparaginase in adults with acute lymphoblastic leukemia. Blood, 135, 987–995.

Apaolaza,I. et al. (2017) An in-silico approach to predict and exploit synthetic lethality in cancer metabolism. Nat. Commun., 8, 1–9.

Lazarus,H. et al. (1969) Nutritional requirements of human leukemic cells. Asparagine requirements and the effect of l-asparaginase. Exp. Cell Res., 57, 134–138. Apaolaza,I. et al. (2017) An in-silico approach to predict and exploit synthetic lethality in cancer metabolism. Nat. Commun., 8, 1–9.

Apaolaza,I. et al. (2019) GMCS: Fast computation of genetic minimal cut sets in large networks. Bioinformatics, 35, 535–537

Li,H. et al. (2019) The landscape of cancer cell line metabolism. Nat. Med., 25, 850–860.

Rancati,G. et al. (2018) Emerging and evolving concepts in gene essentiality. Nat. Rev. Genet., 19, 34–49.

Narta,U.K. et al. (2007) Pharmacological and clinical evaluation of l-asparaginase in the treatment of leukemia. Crit. Rev. Oncol. Hematol., 61, 208–221.

Sykes,D.B. et al. (2016) Inhibition of Dihydroorotate Dehydrogenase Overcomes Differentiation Blockade in Acute Myeloid Leukemia. Cell, 167, 171-186.e15.

Tsherniak,A. et al. (2017) Defining a Cancer Dependency Map. Cell, 170, 564-576.e16.
