# Supplementary material for: A network-based approach to integrate nutrient microenvironment in the prediction of synthetic lethality in cancer metabolism
Source: PLoS Comput Biol. 2022 Mar 14;18(3):e1009395. doi: 10.1371/journal.pcbi.1009395 (PMC8947600; doi:10.1371/journal.pcbi.1009395)
Supplement: Attachment — Submitted filename: ResponseLetter.docx [file pcbi.1009395.s017.docx]

**Reviewer #1:**

With this revision most of the comments have been satisfactorily addressed. A few points require further clarification.

**Response:** We are very grateful for the questions and comments raised by the reviewer. They helped us to significantly improve our manuscript.

Major comments

1) The effect size in supplementary figure 3 looks small based on visual inspection. For a more quantitative argument it would be helpful to also include the correlation coefficient. A low correlation across all expression levels does not detract from the main result, that there are different groups of cell lines that react differently, but if the correlation is low, it would be appropriate to qualify the comments about significant linear relations.

**Response:** We have included the correlation coefficient for both cases in the legend of Supplementary Figure 3. As it was anticipated by the reviewer, we have low linear correlations: 0.097 for cholesterol and 0.154 for myo-Inositol. Thus, we have qualified our comments regarding linear association in the main manuscript. See the last paragraph of Results section (page 16).

2) Supplementary Figure 4, is used as support for a claim of bimodality. For S4a there does indeed seem to be a bimodal distribution such that values < log2(1+1)=1 belong to one distributions and values > 1 belong to another. However, for S4b this is less clear, although the distribution may perhaps be perceived as two overlapping normal distributions with peaks around 3 and 5.5. Here there is no clear distinction for values <1, which may perhaps help explain the lack of significant difference seen between auxotrophic prototrophic in this setting (with exception for the ALL and AML cell lines).

**Response:** The comment from the reviewer is right. Supplementary Figure 4b suggests that the distribution of the expression of the limiting gene for ngMCSs involving myo-Inositol may be bimodal with peaks around 3 and 5.5. The reason of selecting a threshold of 1 TPM for the analysis performed in Figure 4 was merely to apply a conservative strategy in the definition of auxotrophic and prototrophic cell lines.

However, following the reviewer’s comment, we performed the same analysis shown in Figure 4 but selecting as myo-Inositol auxotrophic those cell lines where the limiting gene is expressed less than 3 log_2_(TPM+1) and classifying as myo-Inositol prototrophic those cell lines where the limiting gene is expressed more than 5.5 log_2_(TPM+1). We can observe that the results obtained do not vary at all, indeed, only those ALL and AML auxotrophic cell lines are significantly different to the prototrophic ones. For this reason, we decided to keep our conservative strategy and Figure 4 as presented in the previous version of the manuscript.

****

**

In accordance with this, we hypothesized that the reason underlying the lack of difference between Auxotrophic and Prototrophic cell lines in this setting might be that the inhibition SLC5A3 may be a good proxy of inositol depletion only in the contexts assessed in AML and ALL and that this fact cannot be generalized.

3) The ngMCSs method can be used to predict essentiality of nutrients in cell lines under the assumption that gene expression below a certain value implies lack of flux for its associated reactions. The authors have already done all the work required to perform a global analysis of nutrient essentiality in RPMI culture medium across all cell lines in the CCLE, which would be a binary matrix [nutrients] x [cell lines]. Such a global analysis would help emphasize the generality of the method and additionally may be a useful resource for experimentalist working with these cell lines.

**Response:** We appreciate this comment raised by the reviewer. We have provided the matrix requested by the reviewer in Supplementary Data 2 and added the following sentence in the main text (last paragraph of Results section, page 16):

*“Finally, we provide in Supplementary Data 2 our predictions about which cell lines are dependent on cholesterol, myo-Inositol and the rest of nutrients, following our conservative threshold expression of 1 TPM for the genes involved in ngMCSs.”*

Minor comments

1) The authors comment that “gMCSs of size 1 were not considered in this study” but for the ngMCSs in RPMI culture medium, the first 9 are of size 1 after the revision. The inclusion seems appropriate for completion, but is there a reason for this difference in definition?

**Response:** ngMCS of size 1 define nutrient/genes that are essential for any cell type under RPMI culture medium. These nutrients/genes are essential for all cancer cell lines but also for all normal (healthy) cells, so it is not possible to derive selective (tumor-specific) therapeutic strategies from them. For this reason, we are more interested in investigating context-specific nutrient dependences using gMCSs of size strictly greater than 1, as illustrated in Figure 4. Although it is not developed in this work, we expect higher selectivity of these therapeutic strategies for cancer cells.

2) For supplementary figure 2 and 4 it would be useful to indicate unit for x axis (log2 tpm+1) and y axis (probability density?)

**Response:** We have amended Supplementary Figures 2, 3 and 4 to include this information.

3) The abbreviation ALL for acute lymphoblastic leukemia is explained in the main text but could perhaps also be spelled out in the figure legend, since it can easily be mistaken to mean all cell lines.

**Response:** We have explained all the abbreviations included in the figures in the corresponding figure legends.

4) On page 24 it is written “One continu. One continues”.

**Response:** We have corrected this typo in the main manuscript. We thank the reviewer for all these corrections.

**Reviewer #2:**

I am overall satisfied with the revisions that you have made to the manuscript. The only thing I would still advise that you do is to look more closely at visualizing your results on a pathway map. I still believe this may provide some novel insights, even if it must be restricted only to reactions with a simple reaction-gene relationship. Thank you for your hard work, and good luck with publication.

**Response:** We thank the reviewer for the comments and improvements suggested for our article. As detailed in our previous review, we have explored several visualization tools and ways to project the computed ngMCS onto pathway maps, but state-of-the art methodologies and the obtained results came out to be, not only inefficient, but, in our opinion, provide more confusion than insight to the results. However, we are keen on further exploring or developing such visualization tools in further works.

**Reviewer #3:**

The authors has solved all my questions.

**Response:** We thank the reviewer for all his work and insight during this reviewing process.

**Have the authors made all data and (if applicable) computational code underlying the findings in their manuscript fully available?**

Reviewer #1: Yes

Reviewer #2: Yes

Reviewer #3: **No:** I could not find the detailed analysis codes. Only example codes (MATLAB) were provided.

**Response:** We have included the Matlab and R scripts to perform the analysis and generate the figures together with the Supplementary Material.

REFERENCES

Wei,Y. et al. (2020) In vivo genetic screen identifies a SLC5A3-dependent myo-inositol auxotrophy in acute myeloid leukemia. bioRxiv, 2020.12.22.424018.
